# Targeted Toxicities: Protocols for Monitoring the Adverse Events of Targeted Therapies Used in the Treatment of Non-Small Cell Lung Cancer

**DOI:** 10.3390/ijms24119429

**Published:** 2023-05-29

**Authors:** Jacobi B. Hines, Benjamin Bowar, Emma Levine, Alessandra Esposito, Marina C. Garassino, Christine M. Bestvina

**Affiliations:** 1 Department of Medicine, Section of Hematology/Oncology, University of Chicago Medical Center, Chicago, IL 60637, USA; jacobi.hines@uchicagomedicine.org (J.B.H.); benjamin.bowar@uchicagomedicine.org (B.B.); espositoa@uchicago.edu (A.E.); cbestvina@uchicago.edu (C.M.B.); 2 Booth School of Business, University of Chicago, Chicago, IL 60637, USA; emma.levine@chicagobooth.edu

**Keywords:** non-small cell lung cancer, molecular targeted therapies, adverse drug events, toxicity, pharmacovigilance

## Abstract

Targeted therapies have revolutionized the treatment for many patients with non-small cell lung cancer (NSCLC). Multiple new oral targeted therapies have been approved in the last decade; however, their overall efficacy may be reduced by poor adherence, treatment interruptions, or dose reductions due to adverse events. Most institutions lack standard monitoring protocols for toxicities from these targeted agents. This review describes important adverse events observed in clinical trials and reported by the U.S. Food and Drug Administration for both currently approved and upcoming promising therapies in the treatment of NSCLC. These agents cause a range of toxicities, including dermatologic, gastroenteric, pulmonary, and cardiac toxicities. This review proposes protocols for routine monitoring of these adverse events, both prior to initiation of therapy and while on treatment.

## 1. Introduction

Lung cancer remains the number one cause of cancer-related death worldwide. Non-small cell lung cancer (NSCLC) accounts for most lung cancer diagnoses (84%), and the identification of targetable driver mutations has changed treatment options dramatically over the last decade [1]. More than half of patients diagnosed with NSCLC have an actionable mutation [2]. The identification of driver mutations has resulted in the U.S. Food and Administration’s (FDA) approval of multiple oral and intravenous therapies. The development of oral targeted therapies provides a clear advantage in terms of convenience to the patient but can also result in toxicity and non-adherence [3]. In recent years, the American Society of Clinical Oncology (ASCO) and the Oncology Nursing Society (ONS) jointly published and recently updated guidelines on oral chemotherapy safety standards [4]. Patients receiving these oral anti-cancer therapies appear to have less contact with the treating providers than those receiving intravenous treatments [5]. Given patients receiving targeted agents may have less direct contact with healthcare teams, it is crucial to closely monitor side effects, adherence, and safety. Regular monitoring of oral drugs for cancer is a critical component of comprehensive patient care. It enables healthcare providers to detect and manage potential side effects early, which can help prevent complications, reduce the need for hospitalization, and improve patients’ quality of life. By adjusting treatment as needed, healthcare providers can optimize outcomes and enhance the overall effectiveness of treatment.

There are currently no established guidelines for monitoring toxicities associated with targeted therapy in NSCLC. A survey among cancer centers showed there were limited protocols for monitoring and managing risks associated with targeted therapy [6]. The importance of monitoring has been well established, but there is limited literature that provides monitoring recommendations. This review aims to monitor parameters for targeted agents used for the treatment of NSCLC to improve patient outcomes and tolerability. Below we describe the adverse events for which our recommendations are based. While it is not a fully comprehensive list, we discuss the most common and most severe adverse events (Table 1).

## 2. Literature Search

Our recommendations were based predominantly on FDA prescribing information. We also evaluated landmark trials resulting in FDA approval, trials cited as providing evidence for the National Comprehensive Cancer Network (NCCN) recommendations in the treatment of NSCLC, related papers, and case reports via a PubMed search on 22 October 2022 using the relevant drugs and “adverse events” as keywords without restrictions to assess common toxicities. Toxicities that occurred in more than 10% of the patient population or that were of significant clinical concern in the landmark trials were included in our monitoring parameters. We also incorporated periodic monitoring parameters that were stated in the package insert.

## 3. Results

### 3.1. EGFR Mutation (Exon 19 Deletion or L858R)

#### 3.1.1. First Generation

##### Erlotinib

Originally broadly approved for the treatment of NSCLC in 2004, erlotinib’s indications were later narrowed to first-line, maintenance, or subsequent therapy in patients with EGFR exon 19 deletions or L858R substitutions as a result of the EURTAC and IUNO studies in 2016. The most common toxicities observed in multiple studies included: rash (70–85%), diarrhea (48–62%), and cough (48%). The most common grade 3–4 adverse events included: rash (14%) and dyspnea (8%). Other notable adverse events included: ocular toxicity (12–18%), ILD (1.1%), renal impairment (0.4%), hepatic failure (0.4%), and hemorrhage associated with elevated international normalized ratio (INR) in the setting of concomitant warfarin use [10,31,32].

##### Erlotinib + Ramucirumab

The results of the RELAY study led to the 2020 FDA approval of ramucirumab in combination with erlotinib for the first-line treatment of metastatic NSCLC for patients with EGFR exon 19 deletions or exon 21 L858R mutations. Similar toxicities to erlotinib monotherapy were observed. Adverse events that were described more frequently with the addition of ramucirumab included: hypertension (42%), proteinuria (35%), epistaxis (16%), peripheral edema (13%), and hepatotoxicity (42–43%). Hypertension and diarrhea were the most common grade 3–4 adverse events noted with the addition of ramucirumab (24% and 7%, respectively) [33,34].

##### Gefitinib

Gefitinib was initially approved in the European market in 2009, where it has seen the majority of its use. It has since been approved by the FDA in 2015 for the first-line treatment of metastatic NSCLC with EGFR 19 deletions or exon 21 L858R mutations. The common adverse events of any grade observed in trials included: rash (44.9–66.2%), diarrhea (30.8–44.6%), and nausea (10.3–16.6%) [7,35]. Other notable adverse events of any grade included: proteinuria (35%), ALT (38%), and AST (40%). ILD occurred in 1.3% and ocular disorders occurred in roughly 6.7% of patients. Grade 3–4 adverse events are uncommon with diarrhea (3%) and decreased appetite (2.3%) being reported most frequently [8].

#### 3.1.2. Second Generation

##### Afatinib

Afatinib is a tyrosine kinase inhibitor (TKI) that was first approved in July 2013 as first-line therapy for patients with metastatic NSCLC with EGFR exon 19 deletions or L858R substitutions. In a grouped analysis of the LUX-LUNG 3 and LUX-LUNG 6 trials, dermatologic toxicities, diarrhea, and nail changes were the most common adverse events overall (90%, 96%, and 58%, respectively), as well as the most common grade 3–4 adverse events (15–16%, 15%, and 13–14%, respectively) [36]. Other notable toxicities across all trials included: interstitial lung disease (ILD) in 1.6% of patients, keratitis in 0.7% of patients, and hepatic toxicity in 9.7% of patients [37]. Some online reference guides recommend left ventricular ejection fraction (LVEF) monitoring, though a review of cardiac safety across clinical trials did not show an association with heart failure or a decrease in LVEF [38].

##### Dacomitinib

Dacomitinib gained FDA approval for first-line therapy in metastatic NSCLC with EGFR exon 19 deletion or exon 21 L858R substitution in September 2018 based on the results of the ARCHER 1050 trial. The most frequently observed adverse events of any grade were: diarrhea (87%), paronychia (62%), acne (49%), and stomatitis (44%), with acne and diarrhea as the most common grade 3–4 toxicities (14% and 8%, respectively). ILD occurred in 1.3% of patients [9].

#### 3.1.3. Third Generation

##### Osimertinib

Osimertinib was first approved for the use of T790M mutated NSCLC previously treated with first-generation TKIs after clinical trials demonstrated improved progression-free survival (PFS) (10.1 months vs. 4.4 months) and objective response rate (ORR) (71% vs. 31%) compared to platinum therapy plus pemetrexed [12,13]. The subsequent FLAURA and ADURA trials, respectively, led to the FDA approval of osimertinib in the first-line treatment of metastatic NSCLC and as adjuvant therapy for patients with Stage IB–IIIA EGFR exon 19 deletions or exon 21 L858R mutations. Gastrointestinal and dermatologic toxicities were common in both studies (46–58% and 34–58%, respectively). ILD was observed in 3–4% of patients [39,40]. The FLAURA trial observed QTc changes in 10% of patients receiving osimertinib [39]. A post hoc analysis of the FLAURA and AURA3 trials observed a decrease in LVEF of ≥10 percentage points to an absolute value of <50% in 3.9% of patients [41]. The data did not demonstrate a significant causal relationship, though several recent case reports have observed reversible osimertinib-induced cardiomyopathy [14,15,42]. The most frequent grade 3–4 toxicities included: neutropenia (3.4%), lymphopenia (3.3%), and hyponatremia (3.4%).

### 3.2. EGFR Exon 20

#### 3.2.1. Amivantamab

Amivantamab-vmjw is an EGFR/MET bispecific antibody that was the first FDA-approved treatment for EGFR exon 20 insertion mutated NSCLC. This was a result of the phase I CHRYSALIS study in which the most frequent adverse events of any grade included: rash (86%), infusion-related reaction (66%), paronychia (45%), and hypoalbuminemia (27%). The most commonly reported grade 3–4 adverse events were: hypokalemia (5%), pulmonary embolism (4%), diarrhea (4%), and neutropenia (4%) [43].

#### 3.2.2. Mobocertinib

Mobocertinib is an irreversible oral TKI that was granted accelerated approval for use in previously treated metastatic NSCLC with EGFR exon 20 insertion mutations in 2021. Common clinical adverse events of any grade reported in trials included: diarrhea (82–93%), nausea (30–39%), and rash (33–45%). Other notable toxicities include changes in ALT, AST, or electrolytes in more than 20% of patients. Grade 3–4 adverse events included: lymphopenia (15%) and elevated amylase or lipase (10%) [16,44]. Mobocertinib also carries a boxed warning for QTc prolongation, torsades de pointes, and cardiac toxicity [45].

### 3.3. KRAS G12C

#### 3.3.1. Adagrasib

Adagrasib is an irreversible inhibitor of KRAS that has been granted accelerated approval by the FDA in previously treated KRAS G12C mutated NSCLC. In the KRYSTAL-1 study, adverse events of any grade included diarrhea (70.7%), vomiting (56.9%), serum creatinine increase (34.5%), ALT increase (28.4%), AST increase (26.7%), and hyponatremia (23.3%). Grade 3–4 adverse events included anemia (14.7%), dyspnea (10.3%), pneumonia (12.1%), and QTc prolongation (6.0%) [17].

#### 3.3.2. Sotorasib

Sotorasib, an inhibitor of the RAS GTPase family, was the first FDA-approved therapy for KRAS G12C mutated NSCLC [46]. In CodeBreaK100, a phase II study of sotorasib in previously treated locally advanced or metastatic KRAS G12C mutated disease, the most common treatment-related adverse events were diarrhea (31.7%), nausea (19%), and increase in AST/ALT (15%). Edema of all grades occurred in 13% of patients, and 29% of patients experienced increased urine protein. Hepatotoxicity was the most common grade 3–4 adverse event, occurring in up to 5–6% of patients, and approximately 2.4% of patients experienced grade 3–4 pulmonary toxicities [19].

### 3.4. ALK Rearrangement

#### 3.4.1. First Generation

##### Crizotinib

Crizotinib was the first drug in its class approved for the treatment of patients with metastatic NSCLC with ALK gene rearrangements. Initially approved for ALK gene mutations, crizotinib’s FDA indication has expanded to ROS1 and MET exon 14 skipping mutations. The initial approval for crizotinib was based on a PROFILE 1014 study in which the most common adverse events of any grade included: vision disorders (71%), diarrhea (61%), edema (49%), and vomiting (46%). Notable grade 3–4 toxicities included: hepatotoxicity (14%), neutropenia (11%), fatigue (3%), and dyspnea (3%) [20,47,48].

#### 3.4.2. Second Generation

##### Alectinib

Alectinib is a potent, highly selective second-generation ALK inhibitor that was FDA-approved on 6 November 2017 for the treatment of metastatic NSCLC in patients with an ALK mutation. This approval was given based on the results of the ALEX trial, in which the most common adverse events of any grade included: anemia (20%), peripheral edema (17%), and myalgia (16) [21]. Notable grade 3–4 toxicities included: increased AST (6%), ALT (6%), creatine phosphokinase (CPK) (2.8%), and anemia (1.4%) [49].

##### Brigatinib

Brigatinib is a selective oral TKI that was FDA-approved on 22 May 2020, based on the results of the ALTA-1L trial, in which the most common side effects of any grade included diarrhea (58%), cough (36%), nausea (33%), and hypertension (32%). Notable grade 3–4 toxicities included: elevated CPK (2.8%), lipase (3.7%), amylase (5.5%), ALT (4%), AST (4%), and anemia (3%) [50,51]. The brigatinib dose gradually increased over 2 weeks due to the risk of pneumonitis (5.1%, median onset of 2 days), and for this reason, patients should be assessed on a regular basis [51].

##### Ceritinib

In 2017, ceritinib was FDA-approved for patients with previously untreated metastatic NSCLC with an ALK rearrangement. This was a change from the original indication of patients whose disease had progressed or who were intolerant to crizotinib. This approval was based on the ASCEND-4 trial in which the most common toxicities included: diarrhea, nausea, abdominal pain, vomiting, and fatigue. Notable grade 3–4 toxicities included: ALT elevation (31%), AST elevation (17%), and diarrhea (5%) [18,22]. Similarly, the subsequent ASCEND-8 trial reported that a reduced dose of 450 mg in a fed state resulted in less GI toxicity when compared to a previously approved dose of 750 mg in a fasted state (65.9% vs. 80%), but maintained a relatively high level of ALT elevation (27.3%) [52].

##### Ensartinib

Ensartinib is a potent next-generation ALK inhibitor and has demonstrated 10 times greater potency than crizotinib [53,54]. A phase I/II study demonstrated a considerable increase in PFS with ensartinib compared to crizotinib, as well as a marked improvement in intracranial response [53]. The most common all-grade adverse events were: rash (67.8%), hepatotoxicity (37–48%), and pruritus (26.6%). Notable grade 3–4 toxicities included: rash (11.2%), ALT (4.2%), and edema (2.1%) [53,54].

#### 3.4.3. Third Generation

##### Lorlatinib

Lorlatinib is a third-generation ALK inhibitor with a chemical structure different from other ALK TKIs, designed to cover almost all single resistance mutations emerging after first- or second-generation ALK inhibitors [23]. Lorlatinib received FDA approval on 3 March 2021 based on the CROWN trial. The common side effects noted included: edema (55%), peripheral neuropathy (34%), cognitive effects (21%), diarrhea (21%), and vision disorders (18%) [55]. Notable grade 3–4 toxicities included: hypercholesterolemia (16%), hypertriglyceridemia (20%), weight gain (17%), and hypertension (10%) [55,56].

### 3.5. ROS1 Rearrangement

#### Crizotinib and Entrectinib

These drugs are currently approved for the treatment of ROS1 rearrangements [25,55]. Adverse events are described elsewhere in this manuscript.

### 3.6. BRAF V600E

#### Dabrafenib/Trametinib

The combination of dabrafenib (a BRAF inhibitor) and trametinib (a MEK inhibitor) was approved by the FDA in 2017 based on the results of an open-label, phase II trial demonstrating an overall response rate (ORR) of 63%. The most frequently reported adverse events of any grade included: pyrexia (64%), nausea (54%), fatigue (36%), and peripheral edema (36%). Notable grade 3–4 adverse reactions included: pyrexia (11%), ALT increase (11%), hypertension (11%), and a decrease in LVEF (8%) [57].

### 3.7. NTRK 1/2/3 Gene Fusion

#### 3.7.1. Entrectinib

Entrectinib was FDA-approved on 15 August 2019, based on the results of three multicenter trials for patients with solid tumors and NTRK mutations [58]. An additional benefit of entrectinib is its utility in CNS tumors and CNS metastasis. The most common side effects of any grade in NTRK fusion-positive patients included: dysgeusia (47%), fatigue (35%), diarrhea (29%), and constipation (28%) [58]. Notable grade 3–4 events included: weight gain (7%), anemia (9%), fatigue (5%), hyperuricemia (10%), and cognitive disorders (4.5%). Serious side effects that must be monitored included: congestive heart failure (median onset: 2 months), skeletal fractures (median onset: 3.8 months), QTc prolongation, and hyperuricemia [24,59]. NTRK inhibition causes decreased nociception; as a result, entrectinib also has a unique side effect of withdrawal pain upon discontinuation of therapy that resolves with the resumption of treatment or in a median of 14 days [60].

#### 3.7.2. Larotrectinib

Larotrectinib is a first-in-class highly selective oral TKI that was FDA-approved on 26 November 2018 for adult and pediatric patients with solid tumors with NTRK gene fusions [61]. A recent trial of larotrectinib showed an ORR of 75% amongst patients with NSCLC, which is consistent amongst all solid tumor types [26,62]. The most common toxicities of any grade included: fatigue (37%), nausea (29%), dizziness (28%), cough (26%), increased AST/ALT (45%), constipation (23%), and diarrhea (22%). Notable grade 3–4 toxicities included: neutropenia (2%), anemia (2%), AST, and ALT elevation (3%) [26,62].

### 3.8. MET Exon 14 Skipping

#### 3.8.1. Capmatinib

The results of the GEOMETRY mono-1 trial led to the FDA approval of capmatinib, a MET inhibitor, for the treatment of metastatic NSCLCs with a MET exon 14 skipping mutation. The most reported treatment-related adverse events of any grade included: peripheral edema (51%), nausea (45%), vomiting (28%), and a rise in serum creatinine (24%). More than 20% of patients experienced an increase in amylase or lipase of any grade, and 4.5% of patients experienced ILD/pneumonitis. Grade 3–4 toxicities included: peripheral edema (9%), fatigue (8%), dyspnea (7%), and increased ALT (8%) [27,63].

#### 3.8.2. Crizotinib

While crizotinib is FDA-approved for the treatment of ALK-positive and ROS1-rearranged NSCLC as described above, there is a growing body of evidence that supports its use in MET exon 14 skipping mutations [61,64]. Adverse events in these studies were similar to those reported in other mutations.

#### 3.8.3. Tepotinib

Tepotinib is a selective MET inhibitor that gained FDA approval for use in advanced or metastatic NSCLC harboring MET exon 14 skipping mutation due to the results of the VISION trial. The most common treatment-related adverse events of all grades included: peripheral edema (65%), nausea (26%), and diarrhea (22%) [28]. Notable grade 3–4 adverse events included: increased amylase (4.6%), ALT (4.1%), pneumonia (3.9%), and musculoskeletal pain (2.4%) [65].

### 3.9. RET Rearrangement

#### 3.9.1. Pralsetinib

Pralsetinib was FDA-approved on 4 September 2020 for patients with metastatic NSCLC and RET fusion-positive NSCLC based on the results of the ARROW trial [29]. Common toxicities of any grade included: ILD (10%), HTN (29%), hepatotoxicity (AST 69%, ALT 46%), and hemorrhage (2.5%). Grade 3–4 adverse events included: lymphopenia (20%), neutropenia (10%), and hypertension (14%) [66].

#### 3.9.2. Selpercatinib

Selpercatinib is an oral TKI with potent and selective activity against RET. It was FDA-approved on 21 September 2022, based on the results of the LIBRETTO-001 trial [67]. Common treatment-related side effects of any grade included dry mouth (36%) and diarrhea (25%). Notable grade 3–4 toxicities included: diarrhea (5%), hypertension (19.7%), increased AST (8.8%), and increased ALT (11.4%) [67,68].

### 3.10. ERBB2 (HER 2) Mutation Positive

#### Fam-Trastuzumab Deruxtecan

Based on the results of the DESTINY-Lung01 trial, fam-trastuzumab deruxtecan was FDA-approved on 11 August 2022 for patients with metastatic NSCLC with an ERBB2 (HER 2) mutation [25]. Common toxicities of any grade included: nausea (73%), fatigue (53%), alopecia (46%), neutropenia (35%), and anemia (33%) [69]. Notable grade 3–4 toxicities included: nausea (9%), fatigue (7%), neutropenia (18%), and anemia (10%). ILD is an important severe adverse event, having occurred in 26% of patients in the DESTINY-Lung01 trial [25]. Grade 2 or higher pulmonary toxicities require permanent discontinuation. Additionally, trastuzumab-related cardiotoxicity is a rare but serious side effect. It is recommended to check baseline LVEF periodically during treatment and, if EF < 50%, a different treatment option is recommended [30,69].

## 4. Discussion

The ongoing identification of driver mutations in NSCLC has led to the development of a multitude of targeted therapies which bring new opportunities for treatment but can also cause a considerable degree of morbidity, dose interruptions, and treatment delays. Although the FDA includes recommendations for the monitoring of adverse events in the package insert, the degree to which these are followed is unclear. Through the assessment of clinical trial data, case reports, and other adverse events data provided by the FDA, we have developed much-needed protocols for laboratory assessment and clinical monitoring for the targeted therapies currently used in the treatment of NSCLC.

The ASCO/ONS guidelines highlight the importance of adherence and monitoring of patients on targeted therapy. It is essential to counsel a patient about the importance of adherence and signs or symptoms of toxicity upon initiation of therapy. Studies have shown that roughly half of the patients are fully adherent to prescribed oral chemotherapy regimens [70,71]. One of the major limiting factors to adherence is toxicity, which leads to dose reductions and treatment interruptions [2]. In a survey of 42 national cancer centers, it was established that 88% of adverse events were predictable and 50% were preventable [72]. Early identification of toxicity can lead to improved outcomes, reduced hospitalizations, and better quality of life for patients.

An important aspect of the implementation of these proposed monitoring protocols is the consideration of how to best translate the adverse events described in clinical trials into regular clinical practice. The patients studied in trials are meticulously observed in a manner that may not be feasible or realistic in actual practice. Similarly, patients must meet stringent criteria to allow participation in clinical trials. Investigational trials of new drugs across specialties may exclude 26.3–52.9% of the adult population of interest depending on age and other comorbidities; oncology trials specifically may have a median exclusion proportion of 26.4% [73]. The rates of adverse events in this curated population may not be representative of those in the real world. A retrospective study of oral medications in metastatic renal cell carcinoma demonstrated variability in the rates of adverse events reported in a clinical registry as compared to those in the FDA label and landmark trials [74]. As mentioned above, real-world experience with EGFR inhibitors such as osimertinib suggests additional cardiac toxicities, specifically late toxicities, that were not captured in the landmark trials [14,15,42,75]. It is therefore imperative that we update our screening guidelines to reflect these emerging discrepancies in actual clinical practice.

Another challenge stems from uncertainty about how these new therapies will affect an individual patient and the degree to which toxicity should be expected. Although providers may be inclined to encourage patients to make their own choices regarding different treatment options, based on the patient’s tolerance for different side effects, many patients would prefer to eschew this responsibility and receive a clear recommendation from their provider [76]. Providing clear information to patients about the range of possible effects of therapy is important for establishing perceptions of competent care and building patient trust [77]. Nonetheless, transparent communication can be combined with clear recommendations to ease patients’ psychological burdens.

Our hope is that the standardization of toxicity monitoring will help to minimize and address the above challenges and improve patient outcomes. Implementing and standardizing such a protocol requires a multidisciplinary approach involving collaboration between pharmacists, nurses, and physicians. Pharmacists can provide medication education, monitor for potential drug interactions, and make recommendations to physicians regarding dose adjustments or changes to the treatment plan. Nurses can assist in monitoring for potential side effects and providing ongoing support to patients, while physicians are responsible for prescribing appropriate oral drugs and monitoring patient progress throughout treatment. Similarly, local community and academic centers should work together to align monitoring protocols to help standardize patient care. More data are needed to determine the full extent of adverse events observed in patients longitudinally as well as which patients are more susceptible to certain toxicities in the presence of prior comorbidities.

## Figures and Tables

**Table 1:** Suggested Laboratory and Clinical Monitoring Parameters for Targeted Therapies in NSCLC *.

**1.** **EGFR Mutation (Exon 19 Deletion or L858R)**			
	**Baseline Tests**	**Periodic Tests**	**Symptom Monitoring**
**Erlotinib** (FDA Approval: 16 October 2016)	CMP, INR with concurrent warfarin, eye exam.	CMP, INR with concurrent warfarin: monthly for first 3 months then every 3 months thereafter. Eye exam: reassess at 4–8 weeks after treatment initiation.	Diarrhea (all grades: 48–62%, grade 3 or above: 2–6%); rash (all grades: 70–85%, grade 3 or above: 14%); ILD (all grade: 1.1%) [7].
**Erlotinib +** **Ramucirumab** (FDA Approval: 29 May 2020)	CBC, CMP, BP, eye exam, INR with concurrent warfarin, urinalysis.	CBC, CMP, BP, urinalysis, INR with concurrent warfarin: monthly for first 3 months then every 3 months thereafter. Eye exam: reassess at 4–8 weeks after treatment initiation.	Diarrhea (all grades: 70%, grade 3 or above: 7%); hypertension (all grades: 42%, grade 3 or above: 24%); hepatotoxicity (all grades: 42–43%, grade 3 or above: 5–9%); proteinuria (all grades: 35%, grade 3 or above: 3%); peripheral edema (13%) [8].
**Gefitinib** (FDA Approval: 13 July 2015)	CMP, urinalysis, and INR with concurrent warfarin.	CMP, urinalysis, and INR with concurrent warfarin: monthly for the first 3 months then every 3 months thereafter.	Diarrhea (all grades: 30%, grade 3 or above: 3%); skin reactions (all grades: 47%, grade 3 or above: 2%); ILD (all grades: 1.3%, grade 3 or above: 0.7%); hepatotoxicity (all grades: 38–40%, grade 3 or above: 2%) [9].
**Afatinib** (FDA Approval: 12 July 2013)	CMP, LVEF in high-risk patients per provider discretion.	CMP: monthly for the first 3 months then every 3 months thereafter. LVEF: symptomatic.	Diarrhea (all grades: 96%, grade 3 or above: 15%), cutaneous reactions consisting of rash, erythema, and acneiform rash (all grades: 90%, grade 3 or above: 16%), nail changes (all grades: 58%, grade 3 or above: 13%); hepatotoxicity (all grades: 8–11%, grade 3 or above: 2%, ILD (all grades: 1.5%, grade 3 or above: 1.3%) [10].
**Dacomitinib** (FDA Approval: 27 September 2018)	CBC, CMP, and magnesium.	CBC, CMP, and magnesium: monthly for the first 3 months then every 3 months thereafter.	Diarrhea (all grades: 86%, grade 3 or above: 8%); rash/exfoliate skin reactions (all grades: 78%, grade 3 or above: 21%); ILD (all grades: 0.5%) [11].
**Osimertinib** (FDA Approval: 18 April 2018)	CBC, CMP, ECG, LVEF in high-risk patients per provider discretion.	CBC, CMP: monthly for the first 3 months then every 3 months thereafter. ECG: only in patients with congestive heart failure, electrolyte abnormalities, taking multiple QT-prolonging medications, or a history of prolonged QTc. Monthly for the first 3 months and then every 3 months thereafter. LVEF: every 3 months if risk factors per provider discretion or if symptomatic.	Diarrhea (all grades: 58%, grade 3 or above: 2.2%); rash (all grades: 58%, grade 3 or above: 1.1%); ILD (all grades: 3.9%, grade 3 or above: 0.4%); neutropenia (all grades: 41%, grade 3 or above: 3%); hepatotoxicity (all grades: 21–22%, grade 3 or above: 1%) [12,13].
**2.** **EGFR Mutation (Exon 20 Insertion)**			
	**Baseline Tests**	**Periodic Tests**	**Symptom Monitoring**
**Amivantamab** (FDA Approval: 21 May 2021)	CBC, CMP.	CBC, CMP: every 3 weeks.	Infusion reaction (all grades, any day: 66%; day 1 cycle 1: 65%, day 2 cycle 1: 3.4%, grade 3 or above, day 1 cycle 1 only: 1.8%), ILD (all grades: 3.3%, grade 3 or above: 0.7%); rash (all grades: 74–84%, grade 3 or above: 3.3%) [14].
**Mobocertinib** (FDA Approval: 15 September 2021)	CBC, CMP, amylase, lipase, ECG.	CBC, CMP, amylase, lipase, ECG: monthly for first 3 months then every 3 months thereafter.	Diarrhea (all grades: 93%, grade 3 or above: 21%); ILD (all grades: 4.3%, grade 3 or above: 0.8%); nausea (all grades: 37%. grade 3 or above: 4.4%); elevated pancreatic enzymes (all grades: 35–40%, grade 3 or above: 10–13%) [15].
**3.** **KRAS G12C Mutation**			
	**Baseline Tests**	**Periodic Tests**	**Symptom Monitoring**
**Adagrasib** (FDA Approval: 12 December 2022)	CBC, CMP, amylase, lipase, ECG.	CBC, CMP, amylase, lipase, ECG: monthly for first 3 months then every 3 months thereafter.	Hepatotoxicity (all grades: 32%, grade 3 or above: 1–5%); ILD (all grades: 4.1%, grade 3 or above: 1.4%); diarrhea (all grades: 70%, grade 3 or above: 0.9%); anemia (all grades: 51%, grade 3 or above: 8%); hyponatremia (all grades: 52%, grade 3 or above: 8%) [16].
**Sotorasib** (FDA Approval: 28 May 2022)	CBC, CMP, urinalysis.	CBC and urinalysis: monthly for the first 3 months then every 3 months thereafter. CMP: every 3 weeks for the first 3 months, then monthly thereafter.	Diarrhea (all grades: 31.7%, grade 3 or above: 5%); hepatotoxicity (all grades: 15%, grade 3 or above: 5–6%); ILD (all grades: 0.8%, grade 3 or above: 0.8%); edema (all grades: 15%, grade 3 or above: 0%) [17].
**4.** **ALK Rearrangement**			
	**Baseline Tests**	**Periodic Tests**	**Symptom Monitoring**
**Crizotinib** (FDA Approval: 6 August 2011)	CBC, CMP, ECG.	CBC: monthly for the first 3 months then every 3 months thereafter. CMP: every 2 weeks for the first 2 months, then monthly. ECG: only in patients with congestive heart failure, electrolyte abnormalities, taking multiple QT-prolonging medications, or a history of prolonged QTc. Monthly for the first 3 months and then every 3 months thereafter.	Bradycardia (all grades: 3%, grade 3 or above: 0.6%); diarrhea (all grades: 61%, grade 3 or above: 2%); neutropenia (all grades: 52%, grade 3 or above: 11%); ILD (all grades: 2.9%, grade 3 or above: 0.5%); hepatotoxicity (all grades: 66–79%, grade 3 or above: 7–11%) [18].
**Alectinib** (FDA Approval: 6 November 2017)	CBC, CMP, CPK, BP + HR.	CBC, BP + HR: monthly for the first 3 months and then every 3 months thereafter. CMP: every 2 weeks during the first 3 months then once a month thereafter. CPK: every 2 weeks for the first month and then as needed with symptoms.	ILD (all grades: 0.7, grade 3 or above: 0.4%); renal impairment (all grades: 8%, grade 3 or above: 1.7%); myalgia (all grades: 26%, grades 3 or 4: 0.7%); bradycardia (all grades: 11%, grade 3 or above: 0%); hepatotoxicity (all grades: 40–50%, grade 3 or above: 0–6%); anemia (all grades: 62%, grade 3 or above: 7%) [19].
**Brigatinib** (FDA Approval: 22 May 2020)	CBC, CMP, amylase, lipase, CPK, BP + HR.	CBC, CMP, amylase, lipase: monthly for first 3 months then every 3 months thereafter. CPK: with symptoms. BP + HR: at 2 weeks and monthly thereafter.	ILD (all grades: 3–9%, grade 3 or above: 2.7%); hypertension (all grades: 21%, grade 3 or above: 5.9%); bradycardia (all grades: 5.7%); CPK elevation (all grades: 27%, grade 3 or above: 2.8%); pancreatic enzyme elevation (all grades: 3–45%, grade 3 or above: 4.6–5.5%); hyperglycemia (all grades: 43%, grade 3 or above: 4.6%) [20].
**Ceritinib** (FDA Approval: 26 May 2017)	CBC, CMP, amylase, lipase, ECG, BP + HR.	CBC, amylase, lipase, BP + HR: monthly for first 3 months then every 3 months thereafter. CMP: monthly. ECG: only in patients with congestive heart failure, electrolyte abnormalities, taking multiple QT-prolonging medications, or a history of prolonged QTc. Monthly for the first 3 months and then every 3 months thereafter.	Hepatotoxicity (all grades: 86–91%, grade 3 or above: 21–34%); ILD (all grades: 2.4%, grade 3 or above: 1.3%); hyperglycemia (all grades: 53%; grade 3 or above: 10%); pancreatic enzyme elevation (all grades: 13–37%, grade 3 or above: 6–8%) [21].
**Ensartinib** (FDA Approval: 23 May 2020)	CBC, CMP.	CBC, CMP: monthly for the first 2 months, then every 3 months thereafter.	Congestive heart failure (all grades: 3.4%, grade 3 or above: 2.3%); mood disorders (all grades: 10%, grade 3 or above: 0.6%); dizziness (all grades: 38%, grade 3 or above: 2.2%); hepatotoxicity (all grades: 36–42%, grade 3 or above: 2.5%), anemia (all grades: 67%, grade 3 or above: 9%) [22].
**Lorlatinib** (FDA Approval: 3 March 2021)	CBC, CMP, lipid panel, ECG, BP.	CBC + lipid panel: monthly for the first 2 months, then every 3 months thereafter. CMP: monthly. ECG: only in patients with congestive heart failure, electrolyte abnormalities, taking multiple QT-prolonging medications, or a history of prolonged QTc. Monthly for the first 3 months and then every 3 months thereafter. BP: 2 weeks after initiation and monthly thereafter.	Hepatotoxicity (all grades: 28–37%, grade 3 or above: 1–2%); CNS, including seizures, hallucinations, change in cognitive function and mood (all grades: 54%, grade 3 or above: 1–5%); hyperlipidemia (all grades: 96%, grades 3 or 4: 17); hypertriglyceridemia (all grades: 90%, grade 3 or above: 18%); ILD (all grade 1.5%, grades 3 or 4: 1.2%); anemia (all grades: 52%, grade 3 or above: 4.8%) [23].
**5.** **ROS1 Rearrangement**			
	**Baseline Tests**	**Periodic Tests**	**Symptom Monitoring**
**Crizotinib** (FDA Approval: 11 March 2016)	CBC, CMP, ECG.	CBC: monthly for the first 3 months then every 3 months thereafter. CMP: every 2 weeks for the first 2 months, then monthly. ECG: only in patients with congestive heart failure, electrolyte abnormalities, taking multiple QT-prolonging medications, or a history of prolonged QTc. Monthly for the first 3 months and then every 3 months thereafter.	Bradycardia (all grades: 3%, grade 3 or above: 0.6%); diarrhea (all grades: 61%, grade 3 or above: 2%); neutropenia (all grades: 52%, grade 3 or above: 11%); ILD (all grades: 2.9%, grade 3 or above: 0.5%); hepatotoxicity (all grades: 66–79%, grade 3 or above: 7–11%) [18].
**Entrectinib** (FDA Approval: 15 August 2019)	Uric acid, LVEF in high-risk patients per attending discretion, ECG, lipase, amylase, CMP, CBC.	CMP: every 2 weeks during the first month, then monthly. ECG: only in patients with congestive heart failure, electrolyte abnormalities, taking multiple QT-prolonging medications, or a history of prolonged QTc. Monthly for the first 3 months and then every 3 months thereafter. LVEF: if symptomatic. Uric acid, lipase, amylase, and CBC: monthly for the first 3 months then every 3 months thereafter. Eye exam as clinically indicated.	Congestive heart failure (all grades: 3.4%, grade 3 or above: 2.3%); CNS effects, including dizziness, cognitive impairment, mood disorders, sleep disturbances (all grades: 74%, grade 3 or above: 1%); hepatotoxicity (all grades: 42%, grade 3 or above: 2.5%); hyperuricemia (all grades: 9%, grade 3 or above: 1.7%) [24].
**6.** **BRAF V600E Mutation**			
	**Baseline Tests**	**Periodic Tests**	**Symptom Monitoring**
**Dabrafenib/** **Trametinib** (FDA Approval: 22 June 2017)	CBC, CMP, G6PD, LVEF.	CBC, CMP, LVEF: 1 month after initiation, then every 3 months thereafter. Eye exam and serum glucose/A1c as clinically indicated.	Diarrhea (all grades: 36%, grade 3 or above: 3%); hepatotoxicity (all grades: 11–17%, grade 3 or above: 3–11%); rash (all grades: 22%, grade 3 or above: 3%); hypertension (all grades: 11%, grade 3 or above: 11%), pyrexia (all grades: 64%, grade 3 or above: 11%) [25].
**7.** **NTRK 1/2/3 Gene Fusion**			
	**Baseline Tests**	**Periodic Tests**	**Symptom Monitoring**
**Entrectinib** (FDA Approval: 15 August 2019)	CBC, CMP, uric acid, lipase, amylase, ECG, LVEF in high-risk patients per provider discretion.	CBC, uric acid, lipase, and amylase: monthly for the first 3 months then every 3 months thereafter. CMP: every 2 weeks during the first month, then monthly. ECG: only in patients with congestive heart failure, electrolyte abnormalities, taking multiple QT-prolonging medications, or a history of prolonged QTc. Monthly for the first 3 months and then every 3 months thereafter. LVEF: if symptomatic. Eye exam as clinically indicated.	Congestive heart failure (all grades: 3.4%, grade 3 or above: 2.3%); CNS effects, including dizziness, cognitive impairment, mood disorders, sleep disturbances (all grades: 74%, grade 3 or above: 1%); hepatotoxicity (all grades: 42%, grade 3 or above: 2.5%); hyperuricemia (all grades: 9%, grade 3 or above: 1.7%) [24].
**Larotrectinib** (FDA Approval: 26 November 2018)	CBC, CMP.	CBC: monthly for the first 3 months then every 3 months thereafter CMP: every 2 weeks during the first month, then monthly.	CNS effects, including dizziness, cognitive impairment, mood disorders, sleep disturbances (all grades: 42%, grade 3 or above: 3.9%); hepatotoxicity (all grades: 52%, grade 3 or above: 2.5–3.1%); musculoskeletal pain (all grades: 42%, grade 3 or above: 3.9%); anemia (all grades: 42%, grade 3 or above: 10%); neutropenia (all grades: 36%, grade 3 or above: 14%) [26].
**8.** **MET Exon 14 Skipping**			
	**Baseline Tests**	**Periodic Tests**	**Symptom Monitoring**
**Capmatinib** (FDA Approval: 10 August 2022)	CBC, CMP, amylase, and lipase.	CBC, amylase, and lipase: monthly for the first 3 months then every 3 months thereafter. CMP: every 2 weeks during the first 3 months, then monthly.	ILD (all grades: 4.5%, grade 3 or above: 1.8%); hepatotoxicity (all grades: 13%, grade 3 or above: 6–8%); edema (all grades: 52%, grade 3 or above: 9%); increased pancreatic enzymes (all grades: 26–37%, grade 3 or above: 4–5%) [27].
**Crizotinib** (FDA Approval: 29 May 2018)	CBC, CMP, ECG.	CBC: monthly for the first 3 months then every 3 months thereafter. CMP: every 2 weeks for the first 2 months, then monthly. ECG: only in patients with congestive heart failure, electrolyte abnormalities, taking multiple QT-prolonging medications, or a history of prolonged QTc. Monthly for the first 3 months and then every 3 months thereafter.	Bradycardia (all grades: 3%, grade 3 or above: 0.6%); diarrhea (all grades: 61%, grade 3 or above: 2%); neutropenia (all grades: 52%, grade 3 or above: 11%); ILD (all grades: 2.9%, grade 3 or above: 0.5%); hepatotoxicity (all grades: 66–79%, grade 3 or above: 7–11%) [18].
**Tepotinib** (FDA Approval: 3 February 2021)	CBC, CMP, and amylase.	CBC and amylase: monthly for the first 3 months then every 3 months thereafter. CMP: every 2 weeks for the first 2 months, then monthly.	ILD (all grades: 2.2%, grade 3 or above: 0.9%); hepatotoxicity (all grades: 13%, grade 3 or above: 4.2%); amylase elevation (all grades: 23%, grade 3 or above: 4.6%) [28].
**9.** **RET Rearrangement**			
	**Baseline Tests**	**Periodic Tests**	**Symptom Monitoring**
**Pralsetinib** (FDA Approval: 4 September 2020)	CBC, CMP, BP, ECG.	CBC: monthly for the first 3 months then every 3 months thereafter. CMP: every 2 weeks during the first 3 months then every 3 months thereafter. BP: after 1 week and monthly thereafter. ECG: only in patients with congestive heart failure, electrolyte abnormalities, taking multiple QT-prolonging medications, or a history of prolonged QTc. Monthly for the first 3 months and then every 3 months thereafter.	ILD (all grades: 10%, grade 3 or above: 0.5%); hypertension (all grades: 29%, grade 3 or above: 14%); hepatotoxicity (all grades: 69%, grade 3 or above: 5.4%); neutropenia (all grades: 52%, grade 3 or above: 10%) [29].
**Selpercatinib** (FDA Approval: 21 September 2021)	CBC, CMP, TSH, BP, ECG.	CBC: monthly for the first 3 months then every 3 months thereafter. CMP: every 2 weeks during the first 3 months, then monthly thereafter. TSH: every 6 weeks. BP: after 1 week and monthly thereafter. ECG: only in patients with congestive heart failure, electrolyte abnormalities, taking multiple QT-prolonging medications, or a history of prolonged QTc. Monthly for the first 3 months and then every 3 months thereafter.	Hepatotoxicity (all grades: 45–51%, grade 3 or above: 8–9%); hypertension (all grades: 35%, grade 3 or above: 17%); diarrhea (all grades: 25%, grade 3 or above: 3–5%) [25].
**10.** **ERBB2 (HER 2) Mutation Positive**			
	**Baseline Tests**	**Periodic Testing**	**Symptom Monitoring**
**Fam-Trastuzumab Deruxtecan** (FDA Approval: 11 August 2022)	CBC, CMP, LVEF.	CBC, CMP: on every treatment day. LVEF: every 3 months or if symptomatic.	Nausea (all grades: 73%, grade 3 or above: 9%); neutropenia (all grades: 35%, grade 3 or above: 18%); anemia (all grades: 33%, grade 3 or above: 10%); diarrhea (all grades: 32%, grade 3 or above: 3%) [30].

* This table represents the most common and clinically significant adverse events and is not a fully exhaustive list of potential toxicities.

## Data Availability

No new data were created or analyzed in this study. Data sharing is not applicable to this article.

## Abbreviation

G6PD	Glucose-6-phosphate-dehydrogenase
HR	Heart rate
HTN	Hypertension
INR	International normalized ratio
ILD	Interstitial lung disease
LVEF	Left ventricular ejection fraction
TSH	Thyroid-stimulating hormone
ALT	Alanine aminotransferase
AST	Aspartate aminotransferase
BP	Blood pressure
CBC	Complete blood count
CMP	Complete metabolic panel
CNS	Central nervous system
CPK	Creatine phosphokinase
ECG	Electrocardiogram
